# Delicate dining with a date and burger binging with buddies: impression management across social settings and consumers’ preferences for masculine or feminine foods

**DOI:** 10.3389/fnut.2023.1127409

**Published:** 2023-06-16

**Authors:** Agata Gasiorowska, Michał Folwarczny, Lynn K. L. Tan, Tobias Otterbring

**Affiliations:** ^1^Faculty of Psychology in Wroclaw, SWPS University of Social Sciences and Humanities, Wroclaw, Poland; ^2^Department of Business Administration, Reykjavik University, Reykjavik, Iceland; ^3^School of Social Sciences, Singapore Management University, Singapore, Singapore; ^4^Department of Management, University of Agder, Kristiansand, Norway

**Keywords:** impression management, self-presentation, masculine, feminine, gender image, food preferences, sex differences, mating

## Abstract

Consumers often use their food choices as an impression management strategy to signal desirable aspects about themselves to others, especially in public places like restaurants and cafeterias, where the presence of others can promote certain consumption choices and preference patterns. In mating contexts, people prefer gender-typical traits and characteristics in a potential partner. Food options can also be classified according to their gender typicality, with certain alternatives perceived as feminine (e.g., salad, seafood) and with other options perceived as more masculine (e.g., steak, burger). Drawing on impression management theories from the drinking and dining domain and literature on sex differences in human mate preferences, we present a high-powered experiment investigating whether consumers’ preferences for masculine or feminine foods depend on the social setting in which the food consumption takes place: dining with an attractive date (mating) or meeting and eating with friends (non-mating). Participants (*N* = 162, 46.9% females, 53.1% males; age *M* = 41.8  years, SD = 14.5) were randomly assigned to one of the two experimental conditions (mating vs. non-mating) and were asked to indicate their food preferences for 15 dishes that differed markedly in perceived femininity/masculinity. Consistent with our theorizing, females (males) generally had a stronger preference for foods perceived as more feminine (masculine), thereby supporting the gender-typicality thesis at the aggregate level. Furthermore, females in the mating condition—but not females in the non-mating condition—reported significantly stronger preferences for more feminine food alternatives. However, in direct contrast to our theorizing, males preferred more masculine meals in the non-mating condition (i.e., when dining with friends), whereas this gender-typical tendency did not emerge in the mating condition (i.e., when dining with an attractive date). We discuss the theoretical and practical implications of these findings and present a set of fruitful avenues for future research.

## 1. Introduction

Imagine you are on a date, dining with someone you find attractive. You pick up the menu and browse through the array of food options. Are you more likely to order a beast burger with greasy fries or a slim salad? What about if you were dining, instead, with friends?

Consumption preferences differ depending on who we dine with [e.g., friends vs. family; (1)] and are highly sensitive to social cues in the dining environment, such as the physique of others (2–7) and their physical attractiveness (3, 8, 9). These variations in food preferences are not random, but are driven by specific motives, such as impression management (10–12). An important dimension of food choice in the context of impression management motives is the extent to which a food option signals masculinity or femininity (13). In other words, depending on the social setting, people may have different preferences for foods that are perceived as either masculine or feminine, as choosing such foods may serve as a self-presentation strategy to convey a positive image of oneself to others. In the current study, we sought to examine consumer preferences along the femininity-masculinity continuum in two distinct social contexts: ordering food on a romantic date and eating out with friends. Further, we sought to examine whether this pattern depends on the sex of the consumer (14, 15).

### 1.1. Food choices as an impression management strategy

People are constantly behaving in ways that send signals in social settings to elicit desirable (or prevent undesirable) thoughts or reactions from others (16–18), with this tendency being boosted when the motivation to impress others has been activated (19, 20). One such situation is the date, as the presence of a prospective romantic partner activates mating motives, making people more motivated to engage in impression management to improve their own attractiveness and hence increase their chances on the mating market (21–23). For instance, after viewing images of attractive opposite-sex individuals in “first date” setting rather than stimuli devoid of any romantic connotations, women behave more altruistically (20), with these acts of altruism improving others’ attractiveness judgments of seemingly altruistic targets (24).

Importantly, the strategies of self-presentation to a prospective mate often differ by sex. When a mating motive is active, females are more likely than males to engage in beauty-boosting activities. For example, after seeing pictures of attractive-looking (vs. less attractive) males, females show increased motivation to engage in health-threatening activities (e.g., taking diet pills or tanning) that might amplify their attractiveness (25). Moreover, when females are motivated to outcompete same-sex rivals to get access to mates with good financial prospects (e.g., during a recession), their consumption of beauty-enhancing products increases (26). This urge to signal beauty to prospective mates is also evident in the diet domain. Females prefer to consume lower-calorie foods in the presence of a male (vs. female) dining companion (15); possibly reflecting women’s efforts to convey a desirable impression in the presence of a potential romantic partner. In contrast, males are less concerned with how they present themselves on to potential romantic partners on the healthiness dimension in drinking and dining contexts (27, 28). While women eat significantly less junk food in the presence of an attractive (vs. non-attractive) male companion, men’s junk food consumption appear to be uninfluenced by the attractiveness of their female dining companion (29). Corroborating this finding, exposure to more (vs. less) attractive men decreases women’s willingness to spend money on unhealthy food while increasing their inclination to consume healthy meal alternatives, whereas exposure to more (vs. less) attractive women does not influence men’s drinking and dining decisions between healthy and unhealthy foods, although it increases their desire to acquire expensive foods and beverages (8). Thus, there are clear sex differences in what specific impression management strategies people use after exposure to stimuli that triggers a mating mindset, with females typically displaying “lighter” food consumption preferences (e.g., healthier meal alternatives or food options with fewer calories) and with males preferring pricier food alternatives.

These sex differences are consistent with evolutionary theories of human mate preferences. Males more than females have evolved to prioritize potential mates that are physically attractive, whereas females more than males tend to prioritize status, good financial prospects, and high commitment in a potential mate (30–32), as these strategies optimize reproductive outcomes across the sexes. As such, to improve attractiveness, each sex tends to behave following the mate preferences of the opposite sex, with women who radiate cues of beauty and health having better mate-seeking and reproductive outcomes (33, 34) and with men who embody cues of wealth and status enjoying better mate-attraction prospects (35, 36). Moreover, these strategies to appeal to the opposite sex probably come into play in certain food selection situations. After all, many dates involve food consumption. Hence, in the dating context, females are likely to prefer foods that signal attractiveness and health (e.g., healthier and/or lower-calorie options), while males are more likely to prefer food options that demonstrate their wealth. Such strategies have indeed been shown to be effective. Females are judged as more feminine and less masculine when they eat food with a “good reputation” (e.g., oatmeal with fruits and nuts) compared to females who eat foods with a “bad reputation” (e.g., cake), whereas judgments of males’ masculinity/femininity (as well as other traits such as likability, health, and athleticism) do not differ noticeably depending on what they eat (37). Similarly, females who have eaten smaller (vs. larger) meals are judged by others as more feminine, less masculine, and possessing more gender-typical traits. They are also viewed as having more pronounced appearance concerns and a better physical appearance. At the same time, judgments of males do not differ materially based on their described meal size (27).

The evidence presented above largely focuses on third-party evaluations of consumers’ food choices [e.g., (37, 38)]. In contrast, the current research focuses on first-person preferences in the food domain. Specifically, we test whether and how the social context (mating vs. friendship) may influence consumers’ food preferences. Moreover, while previous literature has investigated whether motives related to mate attraction and affiliation may influence food preferences, such scholarly work has mainly examined consumers’ food choices on the healthiness dimension (healthy vs. unhealthy). As a complement to such prior research, we test how mating and friendship motives may alter food preferences on a different dimension; that is, whether the food itself is perceived as feminine or masculine.

### 1.2. Strategic choices of masculine or feminine foods

People tend to perceive foods on a gender continuum, and this perception may influence their consumption preferences (29, 39). Feminine foods are characterized by smaller portion sizes, elegant presentation styles, and lower calorie content (e.g., vegetables, dairy products, fruits, or fish), whereas masculine foods have larger portion sizes, rough presentation style, and often include meat (e.g., hamburgers or steaks) (40, 41). Across sexes, red meat is generally associated with masculinity, possibly due to the physical strength associated with its acquisition (42, 43), while healthier foods are associated more with femininity (44). When exposed to cues that convey femininity rather than masculinity, men and women are more inclined to prefer healthy foods, while exposure to cues of masculinity mainly motivates consumers across sexes toward unhealthy foods (45). Therefore, because people generally associate foods with femininity or masculinity, these associations can serve important impression management purposes in various social situations. In this study, we focus on the propensity to choose food perceived as feminine or masculine when having a meal with an attractive potential partner and a meal with friends, respectively.

In the context of mating, sexual dimorphism increases attractiveness (46, 47). In other words, to increase their own attractiveness to potential mates, individuals should conform more closely to gender norms. Accordingly, we expect that both sexes should make food choices that are more gender-typical in a dating context relative to a friendship context. Given the link between femininity and perceptions of health (48, 49), which is strongly valued by males in mate selection (30, 31), females should be more inclined to signal femininity in a dating context than in a friendship context. Healthier and lower-calorie foods are judged as more feminine (40), and females prefer lower-calorie foods when dining with a male (vs. female) companion (15). Thus, it is plausible that females are more inclined to choose feminine foods to increase their attractiveness when in a mating mindset. Based on this rationale, we expect that females’ preferences for more feminine foods should be stronger in a dining situation that involves a date with an attractive person rather than meeting and eating with friends.

Using a similar logic, males should be more likely to signal masculinity to enhance their attractiveness in a dating situation when compared to a friendship setting. Although previous studies have found that third-party judgments of females—more than males—are contingent on their food preferences (27, 37), in theory, it is still beneficial for males to strategically present themselves positively in the presence of a prospective mate. Indeed, males expend high levels of cognitive functioning to impress females, whereas this pattern does not emerge to the same extent among females attempting to impress males (50). Thus, regardless of how perceivers judge males’ self-presentation, it is reasonable to expect males to engage in impression management using food choices in a mating context. When males’ gender is emphasized, those who report high conformity to the norms of being a playboy (i.e., a mating motivation) also report lower consumption of vegetables (51), a food perceived as feminine. Presumably, this avoidance of feminine foods strategically improves their attractiveness to females, at least in short-term mating contexts (23). Indeed, when females are asked to rate omnivorous and vegetarian males, they consider omnivorous males more attractive, with this effect mediated by higher levels of masculinity associated with the omnivores (44). Therefore, we expect that men’s preferences for more masculine foods should be stronger when they go out to eat with an attractive date rather than meeting and eating with friends.

## 2. Study overview and research hypotheses

In this study, we present participants with pictures of 15 dishes that differ in terms of femininity and masculinity and ask them for their preferences for these foods in a dating or a friendship context. We used a paradigm proposed by Otterbring et al. (52), in which participants are presented with several cues presumably varying on a certain dimension, and evaluate these cues on this dimension—in this project, the perceived masculinity or femininity of a given meal alternative. A “masculinity index” for each meal option and each participant is then used as a moderator of the effects of interest. We hypothesize that experimentally induced impression management motive via a mating (vs. non-mating) context triggers a stronger preference for gender-typical foods. More specifically, we expected that (H1a) females should have a stronger preference for foods they perceive as more feminine (less masculine), while (H1b) males should be more prone to prefer foods they perceive as more masculine (less feminine), with these presumed effects being particularly pronounced in the mating context (date) compared to the non-mating context (dinner with friends) both for females (H2a) and males (H2b).

## 3. Method

### 3.1. Participants

We recruited 163 US heterosexual participants who reported no dietary restrictions via Prolific Academic; for the purpose of this study, we requested a sex-stratified sample (age *M* = 41.7 years, SD = 14.5, range: [19, 93]; Sex: 46.6% females, 52.8% males, 0.6% did not provide information) to take part in an online study in exchange for 0.75 GBP. We excluded one participant whose self-reported sex was neither female nor male because this cell size was too small for meaningful analyses. The final sample included *N* = 162 (age *M* = 41.8 years, SD = 14.5, range: [19, 93]; Sex: 46.9% females, 53.1% males).

The relevant literature does not provide a meta-analytic effect size for our predicted effects. Moreover, to our knowledge, no studies have been conducted using a similar manipulation as the one designed for the purpose of the current research. Therefore, given the lack of information regarding the expected strengths of our hypothesized effects and the complexity of power simulations for hypothesized interactions in the context of multilevel modeling (53), we decided to conduct a *post-hoc* power analysis instead of an *a priori* power simulation. *Post-hoc* power analysis using the *simr* package for R (54) revealed that our final sample size provided a power of 96% to detect our obtained three-way interaction between femininity/masculinity of the available food options, experimental condition, and participant sex, given our analytic approach, the conventional alpha level of 0.05, and our obtained effect size of *β* = 0.07. The research was conducted in accordance with the World Medical Association Code of Ethics (Declaration of Helsinki) for human experimentation and was approved by the local ethics committee (decision no. 05/P/01/2022).

### 3.2. Stimuli development

Before the main study, we pretested and chose the stimuli for the main investigation. Although there are several existing and validated databases in the literature, such as Food-pics (55), these databases generally show simple images of food against a monochrome background. Therefore, to maximize the ecological validity of our study, we created our own set of food stimuli in a restaurant-like setting that included dishes likely to be served in restaurants.

We downloaded 99 images of complex meals arranged similarly to restaurant dishes from websites that grant an irrevocable, nonexclusive, worldwide copyright license to download, copy, modify, distribute, perform, and use photographs, such as unsplash.com. Forty U.S. participants who did not take part in the main study (age *M* = 35.9 years, SD = 18.0; 45.0% females, 55% males) were recruited through Prolific Academic and rated these dishes in terms of masculinity and femininity using a 201-point scale from −100 = “Very feminine” to 100 = “Very masculine” (*M* = −1.22; SD = 10.93).

We performed multidimensional scaling (MDS) using Jamovi (56, 57). MDS is a multivariate data analysis approach used to visualize the similarity/dissimilarity between the evaluations of objects (here, dishes plotted in a two-dimensional space). We found that the first dimension indeed represented the perceived masculinity or femininity of the foods, while the second dimension seemed to represent whether the dishes presented in the pictures were “ordinary/cheap/canteen style” vs. “classy/expensive/fancy restaurant style.”

Considering that women prioritize cues of status in men (36) and are more inclined to prefer food alternatives with an elegant presentation style themselves (40), males might have been more prone to signal status not only via choosing masculine foods but also through preferences for foods that appear more expensive (8), whereas women could have opted for foods with a classy presentation style rather than food alternatives that mainly conveyed femininity. To control for these confounds, we selected stimuli that differed materially on the first dimension but not considerably on the latter. Therefore, we selected five dishes that participants deemed very feminine (e.g., salads; *M* = −43.74, SD = 30.01, 95%CI [−53.34, −34.15]), five dishes that were evaluated as relatively neutral in terms of their gender image (e.g., tacos and pasta; *M* = 1.97, SD = 26.44, 95%CI [−6.48, 10.43]), and five dishes that were considered very masculine (e.g., steak with fries and burger; *M* = 52.07, SD = 28.89, 95%CI [42.83, 61.31]). All dishes had average scores on the second dimension.

### 3.3. Procedure

Participants were randomly assigned to one of two experimental conditions: an outing with friends (*n* = 84) or a date (*n* = 78). Then, they were asked to perform two tasks presented in a randomized order. In the first task, participants were presented with the 15 pictures of dishes chosen previously and were asked to indicate whether each of the previously rated foods was feminine or masculine using a sliding scale with 1-point intervals (−100 = “very feminine,” 100 = “very masculine”; *M* = 1.07, SD = 10.89). In the second task, participants were asked to imagine going out to dinner with friends (non-mating) or going out to dinner with someone they found attractive for a romantic relationship (mating). To strengthen our manipulation, participants were asked to describe the dinner by writing at least 75 characters (about 12 words) about how they would dress and what such an outing with friends would look like or how the date would be [for a similar procedure, see, e.g., (10, 58–60)]. Next, using a 201-point slider scale, participants had to indicate whether they would order each of the 15 dishes (−100 = “definitely not,” 100 = “definitely yes”). After completing these two tasks, participants provided their demographic data: age and sex.

We refrained from including extensive attention or comprehension checks because we strived to minimize participant fatigue. However, participants were asked to provide a description of an imaginary dinner that was at least 75 characters long. All participants followed these instructions, thus indicating attention. Additionally, participants recruited through Prolific Academic tend to be more attentive and show better task comprehension than individuals recruited through many other crowdsourcing platforms (61, 62). Nevertheless, to further ensure that participants were really paying attention, we embedded a 60-s video showing a cartoon in which an elf gave advice on safe driving in Iceland. Participants were asked to name this character after watching the video. Only four participants were unable to correctly state the character’s name, indicating that they were attentive to the tasks assigned during the experiment.

### 3.4. Analytic approach

Our data were nested because we measured our dependent variable multiple times; that is, each participant was exposed to and rated 15 foods in total. Therefore, we applied linear mixed models to our data using the *lme4* package for R (63). Significance levels were estimated using the *lmerTest* package (64). We used food preferences as the dependent variable (continuous variable ranging from −100 to 100) and the following variables as predictors (fixed effects), including the interaction terms between them: (1) the rated femininity/masculinity of the foods (continuous variable ranging from −100 to 100), (2) experimental condition (z-scored), and participant sex (z-scored). We added random intercepts for participants and food pictures. After testing the three-way interaction model, we decomposed the interactions to formally test our research hypotheses, as described in the results section [for a similar approach, see (65)]. All analyses were performed on standardized (z-scored) continuous variables.

## 4. Results

**Figure 1:**
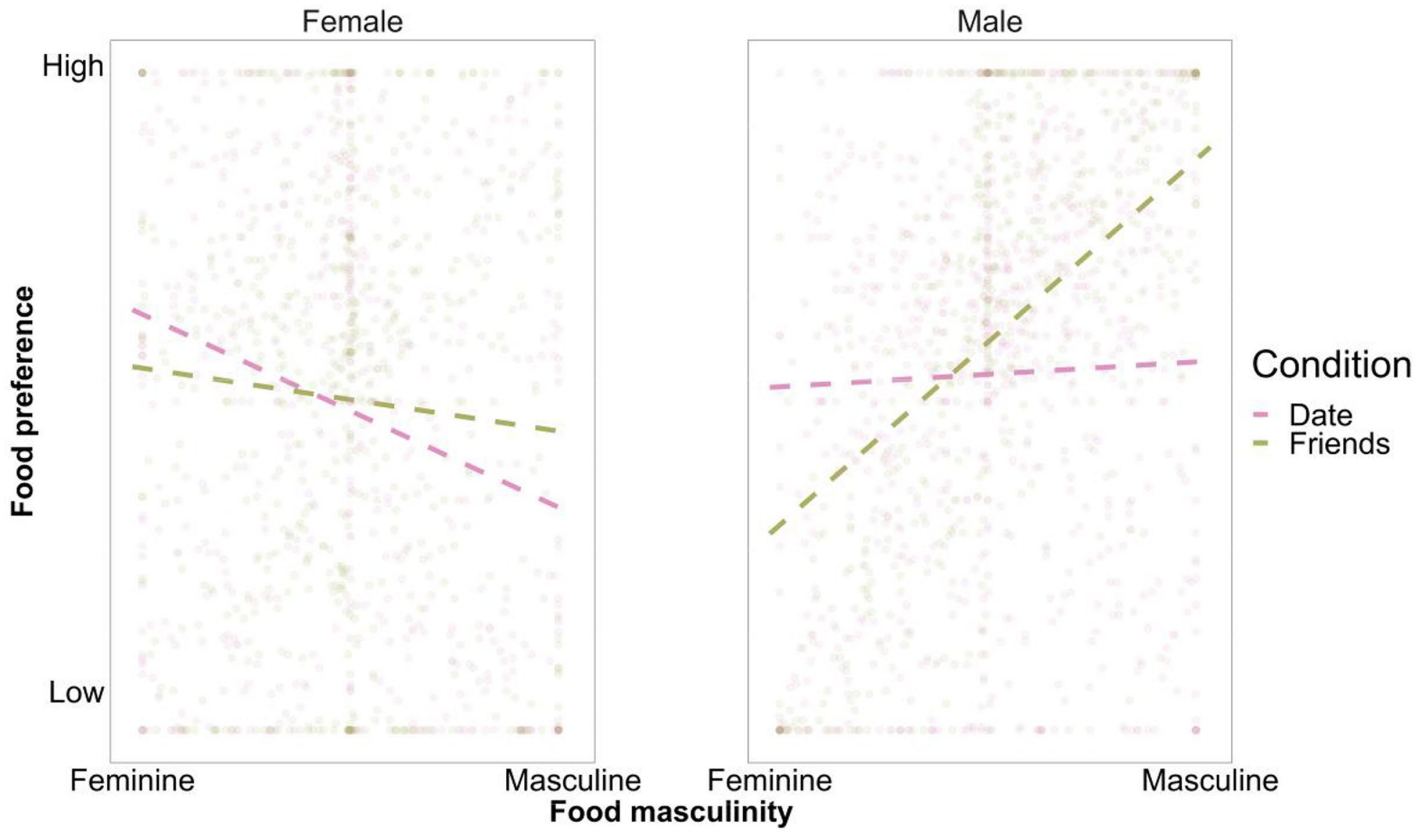
The three-way interaction between participants sex, food masculinity-femininity and social context. The dashed lines represent the regression slopes estimated by fitting linear mixed models to our data. The dots represent individual data points.

To test our main hypotheses, we regressed food preferences on food femininity/masculinity, experimental condition, and participant sex, including all interactions between these three predictors.^1^ Consistent with H1a and H1b, we found a two-way interaction between food femininity/masculinity and participant sex, *β* = 0.21, 95% CI [0.17, 0.24], *p* < 0.001, indicating that males (vs. females) generally preferred more masculine (vs. feminine) foods. Moreover, we found a main effect of food femininity/masculinity, such that more masculine foods were generally more preferred compared to less masculine (more feminine) foods, *β* = 0.06, 95% CI [0.00, 0.12], *p* = 0.033. The main effect of the experimental condition was non-significant, *β* = 0.06, 95% CI [−0.01, 0.12], *p* = 0.083. However, males showed higher food preferences overall than females, *β* = 0.12, 95% CI [0.05, 0.18], *p* < 0.001 (i.e., when asked whether they would order each of the featured dishes, the mean score on the sliding scale was higher for males than for females). We also found a two-way interaction between food femininity/masculinity and experimental condition, *β* = 0.15, 95% CI [0.12, 0.19], *p* = 0.001, such that more masculine foods were preferred to a greater extent when eating with friends than with a date. There was no interaction between experimental condition and participant sex, *β* = 0.03, 95% CI [−0.04, 0.09], *p* = 0.419. Of particular importance for the current investigation, we found a significant three-way interaction between food femininity/masculinity, experimental condition, and participant sex, *β* = 0.07, 95% CI [0.03, 0.10], *p* < 0.001 (see Figure 1).

We decomposed the three-way interaction by performing a simple slopes analysis by participant sex using the *interactions* package for R (66). For females, we found a significant main effect of food femininity/masculinity, *β* = −0.09, 95% CI [−0.17, −0.01], *p* = 0.034, thus further supporting H1a. The main effect of experimental condition was non-significant, *β* = 0.03, 95% CI [−0.07, 0.12], *p* = 0.556. Importantly, we found a significant interaction between experimental condition and females’ food evaluations, *β* = 0.08, 95% CI [0.03, 0.14], *p* = 0.003. For females in the non-mating condition, the slope of food femininity/masculinity was non-significant, *β* = −0.08, 95% CI [−0.16, 0.00], *p* = 0.057. However, consistent with H2a, this effect was negative and highly significant in the mating condition, *β* = −0.24, 95% CI [−0.33, −0.16], *p* < 0.001, suggesting that when females imagined an attractive potential partner, they preferred more feminine (vs. masculine) foods.

For males, in further support of H1b, we found a significant main effect of food femininity/masculinity, *β* = 0.19, 95% CI [0.11, 0.27], *p* < 0.001, while the effect of experimental condition was non-significant, *β* = 0.08, 95% CI [0.00, 0.17], *p* = 0.066. Importantly, we found a significant interaction between experimental condition and males’ food evaluations, *β* = 0.22, 95% CI [0.17, 0.26], *p* < 0.001. For males in the non-mating condition (i.e., those asked to imagine going out to eat with friends), the slope of the femininity/masculinity of the food alternatives was significant and positive, *β* = 0.46, 95% CI [0.38, 0.55], *p* < 0.001, indicating that they showed a stronger preference for masculine relative to feminine foods when going out to eat with friends. However, among males in the mating condition (i.e., those asked to imagine eating with an attractive date), the slope of food femininity/masculinity was non-significant, *β* = 0.03, 95% CI [−0.05, 0.11], *p* = 0.425, suggesting that they showed a similar preference for the available food alternatives, regardless of their perceived femininity/masculinity. This leaves H2b unsupported. Surprisingly, males preferred more masculine meals when dining with friends rather than, as we predicted, with a date.

## 5. Discussion

People change their food preferences depending on the social setting in which meals are consumed (27, 29, 37). In this work, we investigated whether people change their consumption patterns when on a date compared to when they meet and eat with friends. Specifically, we examined how consumer preferences for feminine vs. masculine food options change in each of these settings. Our findings revealed that females (males) reported a stronger preference for foods that they perceive as more feminine (masculine), thus supporting our initial hypotheses (H1a and H1b). We further predicted (as per H2a and H2b) that these gender-typical food preferences should be amplified when a mating (vs. non-mating) motive is activated, such that these preference patterns should be particularly powerful when dining with an attractive date rather than meeting and eating with friends. Supporting H2a, females asked to imagine having a dinner with a date reported significantly stronger preferences for more feminine (less masculine) meal alternatives, with this effect not emerging at the conventional levels of statistical significance among females who were rather asked to dine with friends. These results are consistent with existing theories postulating that sexual dimorphism and the embodiment of gender-typical traits are perceived as more attractive (47, 67). Thus, females seem to strategically seek to enhance their attractiveness to prospective mates by choosing more feminine foods, particularly in mating-relevant contexts.

However, in contrast to H2b postulating that males should prefer more masculine foods when dining with an attractive date rather than with friends, we found the opposite result. Males reported a significantly stronger preference for masculine rather than feminine foods when dining with friends, but not when dining with an attractive date. We elaborate on this unexpected finding below, first commenting on the absence of enhanced preferences for masculine foods among males in the mating context and subsequently on the presence of such preferences in the friendship context.

### 5.1. Why are males not more prone to prefer masculine foods when dining with a date?

The lack of increased preferences for masculine foods among males in the mating context corroborates some findings showing that males are not evaluated based on their food choices to the same extent as females, at least regarding meal sizes and food healthiness. Females who eat healthy rather than unhealthy foods are judged by third parties to be more feminine, likable, healthier, and more athletic, while these social judgments do not differ for males based on what they eat (37). Furthermore, females consuming smaller (vs. larger) meals are evaluated as more attractive, whereas meal size does not affect judgments of males’ attractiveness (27). These results suggest that food choices might be a source of social evaluation of the attractiveness of females more than males, although the attributes to be conveyed by means of one’s meal choices (beauty and health vs. status and wealth) seem to play a sex-differentiated role in shaping such findings (8, 14).

Our results tentatively point to a new angle to understanding male self-presentation strategies to mates, at least in short-term mating contexts (i.e., dates rather than marriage partners). The result that men in our study were more motivated to prefer masculine foods when dining with friends rather than with a date defies theoretical explanations postulating that men are particularly prone to present themselves in gender-typical ways to attract potential partners. Traditionally, the consumption of meat—especially red meat—is seen as a representation of one’s masculine identity in the traditional, “hegemonic masculinity” sense (68). Although “hegemonic masculinity” is broadly accepted as a social norm and is still widely used (69, 70), its validity for describing contemporary masculinity is increasingly being questioned. Recently, Kaplan et al. (71) proposed a measure of “new masculinity,” conceptualized through a set of components such as holistic mindfulness, challenging masculine norms, authenticity, domesticity and caring, and sensitivity to male privilege—most of which has been associated with femininity rather than masculinity. Moreover, De Backer et al. (72) showed that endorsing such a “new masculinity” norm was associated with a weaker attachment to meat, a greater tendency to reduce meat intake, and less negative attitudes toward vegetarians. In addition, excessive meat consumption is harmful to the environment [e.g., (73)], and to human health, particularly men’s health (74), a fact consumers are becoming increasingly aware of. Therefore, our results point to a novel self-presentation strategy that (some) males may undertake to attract mates—they possibly present themselves in “new masculinity” terms and might hence become more concerned about the impact of meat consumption on the environment and their own health in mating contexts (e.g., dining with a date) relative to non-mating contexts (e.g., having a feast with friends). To date, however, these reflections are merely based on *post-hoc* reasoning, underscoring the need for further validation in subsequent research relying on the hypothetico-deductive method, preferably using preregistered designs, predictions, and sample sizes.

Finally, food choices seem more directly associated with health, a highly-valued trait that males seek in females (33). Therefore, the lack of male preferences for more masculine foods that we observed in the mating context may be due to the inability of masculine foods to effectively signal other traits important to females, such as status and wealth (36). Thus, whereas females use food choices as an avenue to improve perceptions of their beauty and health, males might be more strongly evaluated based on cues of status and wealth. It is plausible that males’ food choices influence social judgments of their attractiveness if the foods consumed act as a signal of spending power, as former work suggests (8). Hence, future studies can investigate whether males’ preferences for more expensive drinking and dining options are higher on a date than when they eat out with friends.

### 5.2. Why are males more prone to prefer masculine foods when dining with friends?

In the current research, we hypothesized that males would report stronger preferences for masculine foods when dining with an attractive date rather than friends. We found the complete opposite. Below, we put forth two plausible explanations for this surprising result.

First, compared to females, males are more motivated to achieve, maintain, and demonstrate social dominance, and this social process occurs primarily when males are with same-sex platonic friends as opposed to (opposite-sex) dates (75–77). Moreover, expectations of norm conformity are more stringent for males than females (78, 79), and these norms in male friendships often include displays of dominance. Corroborating this, threats to males’ manhood—but not females’ womanhood—elicits aggressive cognitions (80). It has also been argued that males’ manhood is difficult to earn, yet easily lost, resulting in males (vs. females) facing larger declines in well-being due to the stress of not meeting certain gender expectations (81). Therefore, males in friendships may face social pressure to conform to masculine gender norms (82). Hence, the stronger preferences for masculine foods among males dining with friends might stem from the fact that strict gender-role expectations are experienced by males when they are with their (presumably, same-sex) friends. It seems plausible that this effect should be most prevalent in males whose friends represent a more traditional view of masculinity. Future research should test this possibility.

Second, some men might strategically choose feminine foods on a date to present themselves as prosocial, caring, or environmentally conscious because these traits influence how attractive they are perceived by women (76), and such choices might still be viewed as a failure to meet to “real” masculine standards and threats to their masculinity. Indeed, compared to males whose masculinity is not threatened, males who feel their masculinity is threatened are more likely to (1) consume red meat because they believe eating red meat can improve their masculinity (83), (2) report higher strength abilities during gym exercises (84), (3) avoid purchasing products with a feminine connotation, such as engaging in green consumption (85), (4) choose more masculine beverages (86), and (5) eat more meat pizza than vegetarian pizza (87). If females expect modern males to be masculine in a “new” rather than a “hegemonic” way, and if males mirror those expectations to impress females in the mating context, then men might use masculine foods as a compensatory strategy to “redeem” their masculinity in the face of their peers. Future research should examine whether males have higher expectations of adherence to traditional gender norms when they are with friends than when they are dating, and whether males perceive adherence to non-traditional norms in a mating context as a threat to their masculinity that must be compensated for subsequently.

### 5.3. Limitations

There are several limitations in our research that can be improved in future work. First, the study was based on data from a Prolific Academic sample and relied solely on self-report. Although the data quality from online labor markets has been questioned, research shows that self-report data collected on Prolific Academic, if anything, are superior to similar data collected using traditional methods and other crowdsourced online platforms (61, 62). However, while self-report measures such as hypothetical choice, willingness to pay, and behavioral intentions are common in this stream of research and have been found to produce comparable results as those obtained for behavioral variables [e.g., (3)], it remains to be tested to what extent our results reflect the actual attitudes, judgments, or preferences of our participants. Therefore, despite the fact that we sought to boost the realism of our work by using real food images, as such procedures can compellingly enhance the ecological validity of lab-based experiments (88, 89), future studies should optimally collect data from participants who are not members of online panels, and use behavioral measures and real-life settings to test the effects examined herein (90–93).

Second, we did not account for individual differences that may have influenced our results. Specifically, factors such as the tendency for strategic impression management (94), acceptance of traditional (vs. nontraditional) gender norms or ideologies (71), and the perception of one’s own femininity/masculinity might moderate our obtained results.

Third, our study was restricted to US participants, while preferences for femininity and masculinity in potential mates, and hence the propensity to signal these traits through food choices, might depend on specific ecological conditions that differ across countries and continents (95, 96). For example, prior work has revealed that females prefer more masculine males (67), whereas males prefer more feminine females in countries with better health indices and economic circumstances (46). In contrast, males prefer masculine traits in females under adverse and harsh conditions, where scarcity is prevalent, because masculinity is associated with higher dominance and the ability to acquire resources necessary for survival (46). This pattern demonstrated for country-level data has also been observed at the individual level: after exposure to cues of food scarcity, people generally prefer masculine and calorie-dense foods (40, 41, 97). Therefore, future research should examine how the individual experience of financial threat/hardship (98) and real or anticipated food scarcity/insecurity (97, 99, 100) may increase the tendency to choose masculine foods to enhance attractiveness perceptions and enhance people’s prospects on the mating market.

Fourth, as we sought to rule out presentation style as a design flaw and hence a potential confound to the masculinity or femininity of participants’ preferred food options, we opted for solely choosing food images that did *not* differ materially in terms of their presentation style. While this decision arguably created a “cleaner” dependent variable that was not confounded by other important dimensions—providing rigor, control, and internal validity—this design decision may also have resulted in lower realism and external validity. Therefore, mixing methods might be important in future research on this topic to maximize not only internal validity but also to boost realism, external validity, and real-world applicability of the theorizing underlying the current research.

Finally, as in previous related research [e.g., (9, 20, 23)], our study refers to heterosexual mating and relationships. Therefore, considering that we refer to specific types of romantic relationships and binary conceptualization of sex and gender, our results are restricted to those approximately 96 ± 2% of the population whose sexual orientation can be described as heterosexual (101–103). Accordingly, our results cannot necessarily be generalized to the remaining 4 ± 2% of the population, characterized by other sexual orientations, gender identities, and relationships.

## 6. Conclusion

The current study shows that gender and social context are important factors that determine impression management strategies through food preferences. We demonstrate that females (males) prefer more feminine (masculine) food choices in general. Importantly, females are particularly prone to prefer feminine foods when dining with an attractive date but not when they eat with friends. In contrast, men are materially more inclined to prefer masculine foods when eating with friends but not when dining with a date.

## Data availability statement

The raw data supporting the conclusions of this article will be made available by the authors, without undue reservation.

## Ethics statement

The studies involving human participants were reviewed and approved by the local ethics committee (decision no. 05/P/01/2022). The patients/participants provided their written informed consent to participate in this study.

## Author contributions

All authors listed have made a substantial, direct, and intellectual contribution to the work and approved it for publication. The order of authorship was determined by the authors’ impression management motivation, after adjusting for their meat preferences.

## Funding

This project was financed by a grant from the Institute of Psychology, SWPS University of Social Sciences and Humanities, awarded to AG.

## Conflict of interest

The authors declare that the research was conducted in the absence of any commercial or financial relationships that could be construed as a potential conflict of interest.

## Publisher’s note

All claims expressed in this article are solely those of the authors and do not necessarily represent those of their affiliated organizations, or those of the publisher, the editors and the reviewers. Any product that may be evaluated in this article, or claim that may be made by its manufacturer, is not guaranteed or endorsed by the publisher.

## Footnotes

^1^Participant age did not moderate the impact of experimental condition or participant sex (or their interaction) on preferences for masculine (vs. feminine) foods, thus attesting to the generalizability of our main findings.
